# Temporal and Pharmacological Characterization of Angiostatin Release and Generation by Human Platelets: Implications for Endothelial Cell Migration

**DOI:** 10.1371/journal.pone.0059281

**Published:** 2013-03-15

**Authors:** Aneta Radziwon-Balicka, Cesar Moncada de la Rosa, Barbara Zielnik, Adrian Doroszko, Paul Jurasz

**Affiliations:** 1 Faculty of Pharmacy and Pharmaceutical Sciences, University of Alberta, Edmonton, Alberta, Canada; 2 Department of Pharmacology, Faculty of Medicine and Dentistry, University of Alberta, Edmonton, Alberta, Canada; 3 Cardiovascular Research Centre, University of Alberta, Edmonton, Alberta, Canada; 4 Department of Internal Medicine, Occupational Disease and Hypertension, Wroclaw Medical University, Wroclaw, Poland; Center for Cancer Research, National Cancer Institute, United States of America

## Abstract

Platelets play an important role in thrombosis and in neo-vascularisation as they release and produce factors that both promote and suppress angiogenesis. Amongst these factors is the angiogenesis inhibitor angiostatin, which is released during thrombus formation. The impact of anti-thrombotic agents and the kinetics of platelet angiostatin release are unknown. Hence, our objectives were to characterize platelet angiostatin release temporally and pharmacologically and to determine how angiostatin release influences endothelial cell migration, an early stage of angiogenesis. We hypothesized anti-platelet agents would suppress angiostatin release but not generation by platelets. Human platelets were aggregated and temporal angiostatin release was compared to vascular endothelial growth factor (VEGF). Immuno-gold electron microscopy and immunofluorescence microscopy identified α-granules as storage organelles of platelet angiostatin. Acetylsalicylic acid, MRS2395, GPIIb/IIIa blocking peptide, and aprotinin were used to characterize platelet angiostatin release and generation. An endothelial cell migration assay was performed under hypoxic conditions to determine the effects of pharmacological platelet and angiostatin inhibition. Compared to VEGF, angiostatin generation and release from α-granules occurred later temporally during platelet aggregation. Consequently, collagen-activated platelet releasates stimulated endothelial cell migration more potently than maximally-aggregated platelets. Platelet inhibitors prostacyclin, S-nitroso-glutathione, acetylsalicylic acid, and GPIIb/IIIa blocking peptide, but not a P2Y12 inhibitor, suppressed angiostatin release but not generation. Suppression of angiostatin generation in the presence of acetylsalicylic acid enhanced platelet-stimulated endothelial migration. Hence, the temporal and pharmacological modulation of platelet angiostatin release may have significant consequences for neo-vascularization following thrombus formation.

## Introduction

Platelets are well known to contribute to the promotion of new blood vessel growth and do so by releasing a large repertoire of angiogenesis promoting factors largely from their α-granules [1], [2]. Included amongst these angiogenesis promoters is vascular endothelial growth factor, one of the most potent endothelial cell growth and survival factors [3], [4]. To counter-balance such potent angiogenesis molecules, platelets also release factors that limit new blood vessel growth including the angiogenesis inhibitor angiostatin [5]. Angiostatin is a proteolytic fragment of plasminogen containing the first four-kringle subunits (K1–4). It was first discovered in a mouse Lewis Lung carcinoma model of concomitant resistance [6]. In addition to being formed by cancer and inflammatory cells [7], angiostatin is also present in healthy humans. It is found in abundance in human plasma [8], and it is constitutively generated by platelets and released in active form upon aggregation [5], [8]–[9].

Angiostatin suppresses angiogenesis by inhibiting endothelial cell proliferation, [10], [11], migration [12]–[14], and can even promote endothelial apoptosis [15]–[17]. Recently, we have demonstrated that angiostatin in concentrations generated by platelets inhibits endothelial migration an important early step of angiogenesis by inhibiting matrix metalloproteinase-2 and -14 expression [18]. Moreover, this inhibition in MMP-dependent endothelial cell migration only occurs in hypoxic microenvironments such as would occur following platelet thrombus formation. Because many anti-platelet factors and agents prevent platelet aggregation and thrombus formation, we investigated the effects of pharmacological platelet inhibitors on angiostatin release and generation. Moreover, because thrombus formation can lead to hypoxia, we further investigated the effects of pharmacological platelet inhibitors on platelet-stimulated endothelial cell migration during hypoxia. We hypothesized that platelet inhibitors, in addition to inhibiting release of pro-angiogenic factors, would inhibit angiostatin release, but not generation. This would then result in reduced endothelial cell migration. In addition, because platelet aggregation and thrombus formation occur in a coordinated series of events over time, we characterized temporally platelet angiostatin release, and its effects on endothelial cell migration.

## Materials and Methods

### Reagents

Human plasma-isolated angiostatin was obtained from Pierce Biotechnology (Rockford, IL, USA). Anti-angiostatin antibody (AF226) was obtained from R & D Systems (Minneapolis, MN, USA). Anti-VEGF antibody (Ab-7) was obtained from Lab Vision (Fremont, CA, USA). Anti-P-selectin antibody (clone AK4) was obtained from BD Biosciences (Mississauga, ONT, Canada). 6 nm anti-goat IgG gold and 12 nm anti-mouse IgG gold antibodies were obtained from Sigma (Mississauga, ONT, Canada). Anti-mouse R-phycoerythrin and anti-goat FITC 488 were obtained from Jackson ImmunoResearch Inc (West Grove, PA). Prostacyclin-sodium salt, S-nitroso-glutathione, acetylsalicyclic acid, MRS2395, RGDS peptide, and aprotinin were obtained from Sigma (Mississauga, ONT, Canada). Unless otherwise specified all other reagents were obtained from Sigma.

### Blood Platelets and Platelet Aggregation

Approval for the current study was obtained from the University of Alberta Human Research Ethics Board. Following written informed consent, blood was collected from mixed healthy volunteers who had not taken any drugs for 14 days prior to the study. Prostacyclin-washed platelets were prepared as described previously in Tyrode’s buffer [5], [9]. Platelet samples were pre-incubated for 2 minutes at 37°C in a lumi-aggregometer (Chronolog, Havertown, PA) with vehicle controls or anti-platelet agents. Platelet aggregation was initiated by collagen (3 µg/ml) and monitored by Aggro-Link software for 6 minutes as previously described [5]. After aggregation, platelet pellets were separated from releasates using centrifugation (1000 *g* for 10 minutes). Platelet pellets and releasates were then stored at −80°C for further use [8]. In platelet aggregation experiments where angiostatin generation was inhibited by aprotinin, equal concentrations of aprotinin were supplemented in control releasates following aggregation but prior to releasates storage at −80°C [9]. This was done to negate any potential effects aprotinin may have in endothelial cell migration assays performed in response to platelet releasates. In addition, in some experiments acetylsalicylic acid (ASA) (100 µM) was incubated with isolated platelet rich plasma (10 min), and then prostacyclin-washed platelets were prepared. This permanently inhibited platelet cyclooxygenase activity, but assured that ASA was not present in the Tyrode’s buffer.

### Immunoblot

Immunoblots of platelet pellets and releasates were performed as previously described [5]. 40 µg per lane of platelet protein was loaded prior to SDS-PAGE. Blots were blocked overnight and then incubated with goat anti-human angiostatin (0.8 µg/ml) (R&D Systems) for 2 hours. Anti-goat (0.8µg/ml) horseradish peroxidase–conjugated antibodies were used as the secondary antibodies (Sigma, Oakville, ON, Canada). Immunoreactive bands were visualized with ECL Plus (Amersham Biosciences, San Francisco, CA). Blot bands were quantified using a VersaDoc MP5000 molecular imager with Quantity One software (Bio-Rad, Mississauga, ON, Canada). Results were expressed as arbitrary units of density per mg protein.

### ELISA

A VEGF ELISA (R&D Systems; Minneapolis, MN, USA) was performed to quantify VEGF release by human platelets [8].

### Cell Culture

Cardiac-derived human microvascular endothelial cells (HMVEC-C) were obtained from Lonza (Walkersville, MD, USA). HMVEC-C were cultured in a humidified atmosphere at 37°C and 5% CO_2_ in EGM-2 MV. Cells were supplied with fresh medium every 2 days and sub-cultured upon reaching confluence.

### Immunogold Electron Microscopy

Immunogold electron microscopy was carried out as described previously [19]. Briefly, platelets were fixed in a mixture of 2% glutaraldehyde and 2% paraformaldehyde in 0.1 M phosphate buffer, pH 7.4 for 30 min at 21 ^o^C. Samples were then embedded in LR gold resin (Polysciences Inc, Warrington, U.S.A.) and thin sections were cut and mounted on uncoated nickel grids. For cytochemical staining the following antibodies were used: monoclonal anti-P-selectin, (BD Biosciences) and polyclonal anti-angiostatin (R & D Systems). Following double labelling with anti-P-selectin (1∶25 v/v) or anti-angiostatin antibodies (R&D Systems) (1∶1000 v/v), the samples were treated with mouse (P-selectin immunodetection) and goat (angiostatin immuno-detection) anti-IgG-gold (12 and 6 nm, particle size). In control experiments the primary antibodies were omitted. Platelets were examined with a Philips 410 electron microscope.

### Confocal Microscopy

For confocal immunofluorescence microscopy platelets were fixed for 20 minutes in 4% formaldehyde in Tyrode’s buffer. Solutions of fixed platelets were cytospinned onto polylysine-coated coverslips at 250 g for 5 minutes. Platelets were permeabilized with Tyrode’s Buffer containing 0.1% Triton X-100 (30 min). Specimens were blocked in phosphate-buffered saline (PBS) with 5% BSA for 2 hours, followed by incubation with anti-angiostatin antibodies (R&D Systems) (1∶100 dilution) for 2 hours. Coverslips were washed 3x with PBS and treated with appropriate secondary antibody for 2 hours, and then washed 3x with PBS. Subsequently, coverslips were incubated with anti-VEGF antibody (1∶50 dilution) in the manner described above. Controls were treated in the same fashion except for exclusion of the primary antibody. Preparations were mounted in Prolong Gold Antifade solution (Invitrogen) and analyzed at room temperature on a Leica TCS SP5 microscope equipped with a 100×/1.4 NA objective. Electronic shutters and image acquisition were under the control of Leica LAF AS software.

### Migration Assays

MMP-dependent migration assays were performed as described previously [18]. Briefly, cell culture inserts with 8 µm pores (BD Falcon™) were coated with gelatin (1 mg/ml) (Sigma) for 2 hours at 37°C. After 2 hours, excess gelatin was removed, the inserts were washed 1x with 100 µl of sterile PBS, and allowed to air dry for 30 min. HMVEC-C were serum starved in EBM-2 medium (Lonza) supplemented with 0.5% FBS overnight. HMVEC-C were detached using 1 ml of Trypsin-EDTA solution (Lonza). The trypsin was inactivated using 10 ml of EBM-2+0.5% FBS and HMVEC-C pelleted. The cells were washed 1x, resuspended in EBM-2+0.2% FBS, and counted. 1×10^4^ HMVEC-C in 500 µl were added to each insert and allowed to migrate toward a gradient formed by adding isolated platelet releasates to the wells of insert companion plates. Plates and inserts were incubated at 37°C in a humidified atmosphere under hypoxic conditions, which were generated in a Billups-Rothenberg chamber continuously gassed with 95% N_2_-5% CO_2_. After 24 hours, non-migrating HMVEC-C were removed by scrubbing the upper surface of the inserts with a cotton-tipped swab. The cells on the lower surface membrane were fixed in 4% formaldehyde in PBS and stained with Diff-Quik stain. The membrane was examined by light microscopy using an Olympus CKX41 microscope (Olympus America Inc., Melville, NY) equipped with an *Infinity 1* digital camera. Photomicrographs were captured at the top, bottom, right, left, and center of each insert. Each field of view was counted using ImageJ software, and the results were expressed as the average number of HMVEC-C migrated per field of view.

### Statistics

Statistics were performed using Graph Pad Prism 3.0 software. All means are reported with SE. One-way ANOVA with Tukey’s multiple comparisons test and paired Student’s T-tests were performed where appropriate. A P-value less than 0.05 was considered as significant.

## Results

### A Temporal Difference Exists between Angiostatin and VEGF Release from Platelets

**Figure 1:**
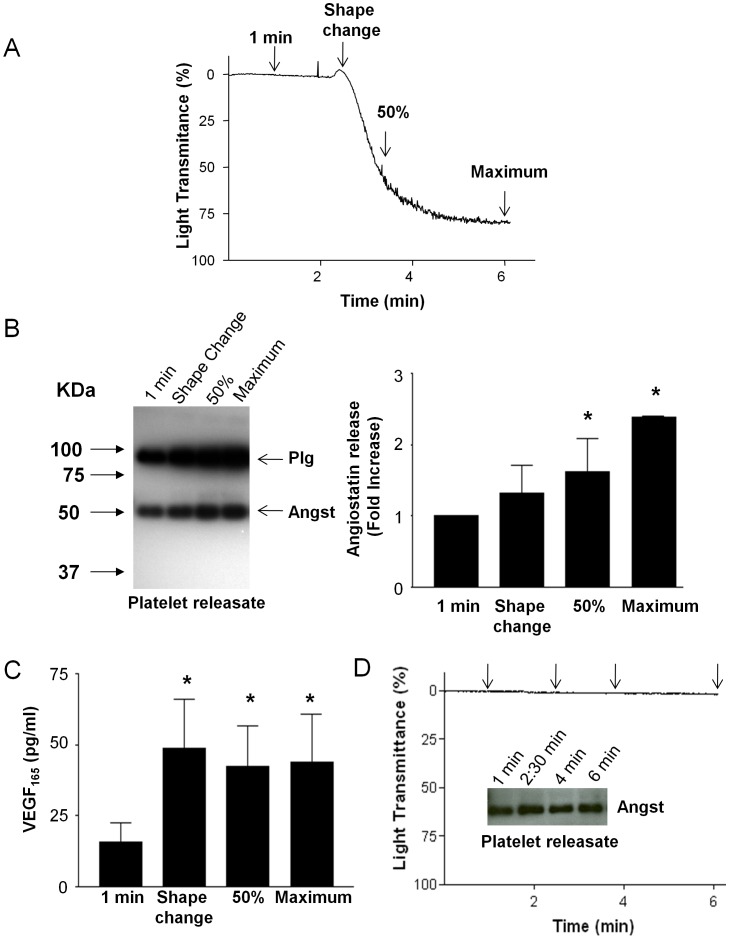
Temporal angiostatin release from platelets. (**A**) Represenative platelet aggregation trace. Collagen (3 µg/ml) was added at time 2 minutes. Arrows indicate time points at which platelet releasates were sampled. (**B**) Represenative angiostatin immunoblot and summary data. N = 4. (**C**) Summary data of platelet VEGF release. (**D**) Represenative platelet aggregometry trace and angiostatin immunoblot demonstrating that in absence of collagen passive release of angiostatin does not significantly occur over 6 minutes. N = 4. *, P<0.05 vs. time 1 minute. Angst – Angiostatin.

Human platelets were incubated in an aggregometer and platelet releasates were sampled at the following time points: one minute prior to the addition of collagen, during platelet shape change following addition of collagen, at 50% light transmittance, and maximal aggregation (Fig. 1A). During each of these time points angiostatin and VEGF release was assayed. Compared to the passive release of angiostatin from unactivated platelets (1 minute time point), angiostatin release from collagen-stimulated platelets followed that of aggregation with maximum angiostatin release occurring upon maximum aggregation (Fig. 1B). In contrast, the release of the pro-angiogenic molecule VEGF from platelets occurred much earlier during platelet activation by collagen with maximum release occurring during platelet shape change (Fig. 1C). In absence of collagen to stimulate aggregation, passive platelet release of angiostatin did not significantly increase over 6 minutes (Fig. 1D).

**Figure 2:**
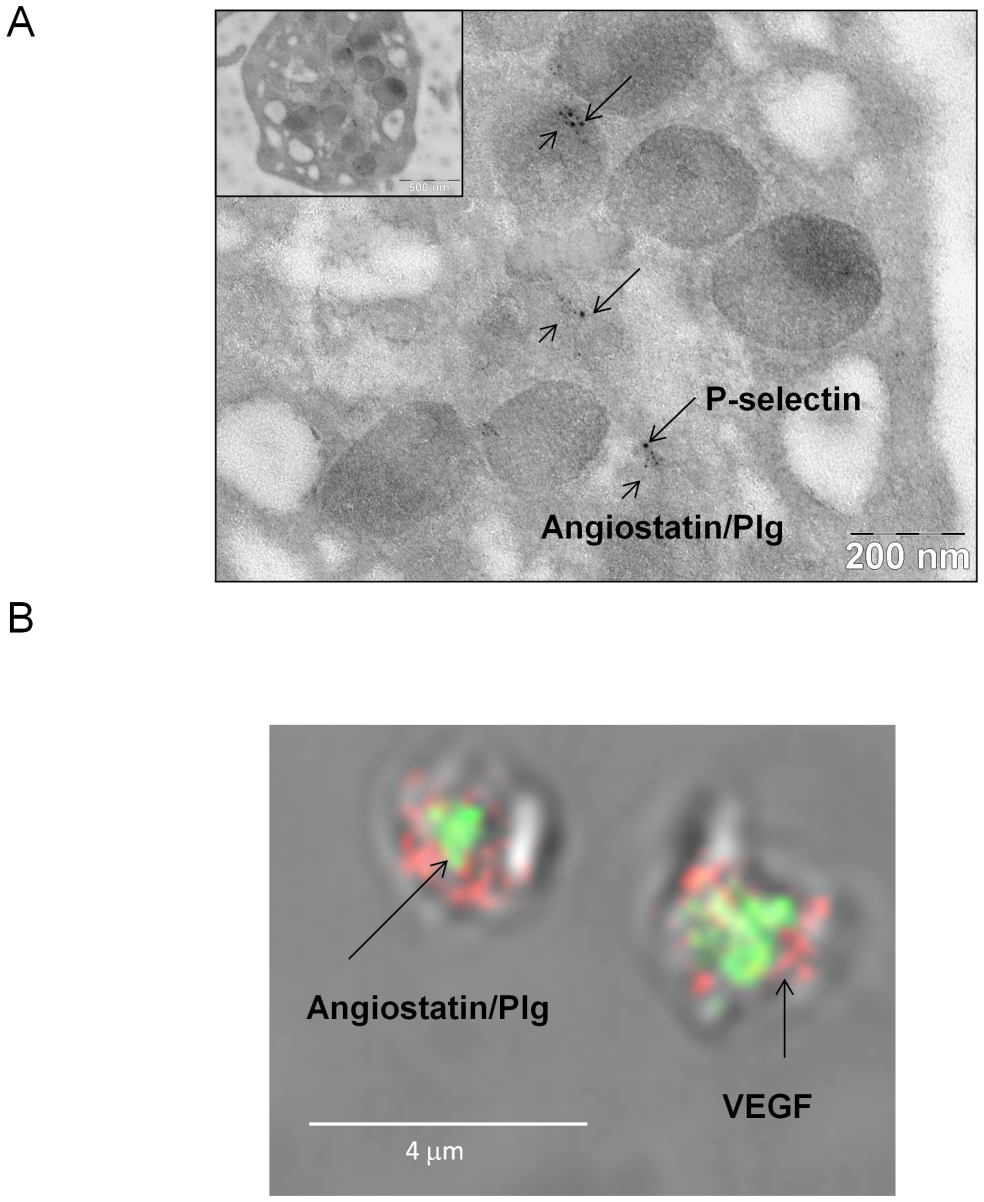
Localization of angiostatin to platelet α-granules. (**A**) Represenative platelet immunogold-electron microscopy and (**B**) confocal immunofluorescence microscopy. N = 3.

Previous studies have shown that in platelets pro-angiogenic factors such as VEGF are stored in either distinct subsets of α-granules or α-granules compartments from that of anti-angiogenic factors such as endostatin [20]–[22]. Hence, we hypothesized that perhaps the differential storage of angiostatin from that of VEGF may explain the differences in temporal release of the two angiogenesis regulators. First, we performed immunogold electron microscopy to confirm whether angiostatin like VEGF is stored in α-granules. Indeed, angiostatin/plasminogen immunoreactivity strictly co-localized in platelet granular structures with P-selectin. Thus, angiostatin, like VEGF is stored in platelet α-granules (Fig. 2A). Next, immunofluorescence confocal microscopy revealed that although both angiostatin and VEGF are stored in α-granules they are segregated in either distinct α-granule subpopulations or compartments, since angiostatin and VEGF immunoreactivity did not co-localize (Fig. 2B)

**Figure 3:**
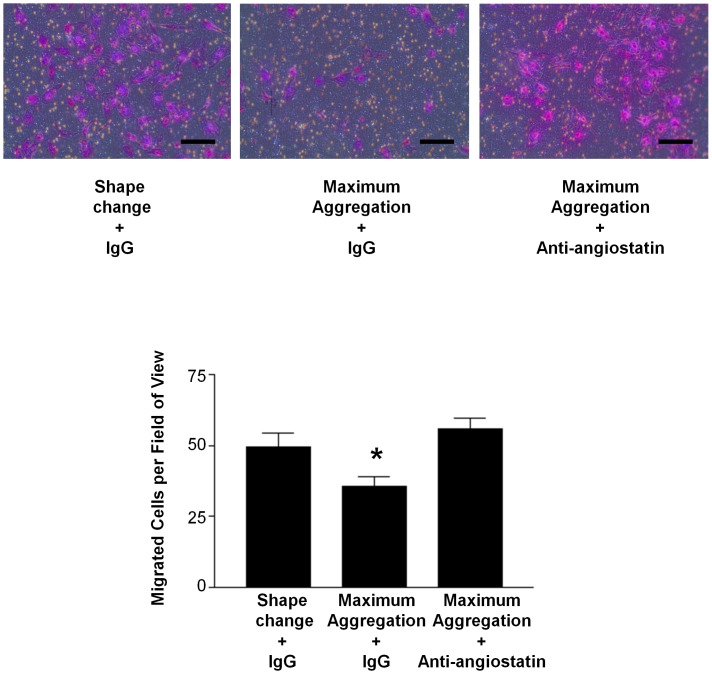
Angiostatin suppresses platelet-stimulated endothelial cell migration. Representative HMVEC-C migration photomicrographs and summary data. HMVEC-C migration in response to platelet releasates from collagen (3 µg/ml) activated (during platelet shape change) and maximum aggregated platelets under hypoxic conditions. Scale bars = 100 µm. N = 4. *, P<0.05 vs. control.

To determine the functional significance of this temporal difference in angiostatin versus VEGF release from platelets, we performed endothelial cell migration assays in response to platelet releasates isolated during shape change versus those isolated upon maximal aggregation. Endothelial cell migration is an important early stage of angiogenesis, and platelets under normoxic conditions provide a powerful stimulus for endothelial cell movement (Figure S1). Moreover, because pathologically platelet aggregation/thrombus formation often results in hypoxia, we performed the migration assays under hypoxic conditions with cardiac-derived human microvascular endothelial cells (HMVEC-C). Platelet releasates isolated during shape change were more potent at stimulating HMVEC-C migration than releasates isolated following maximal aggregation (49.5±4.7 vs. 35.8±3.1 cells per field of view, *P<0.05*) (Fig. 3). This weaker ability of maximal aggregation releasates to stimulate migration was a direct result of increased angiostatin levels in these releasates, as angiostatin inhibition with a neutralizing antibody [5] restored HMVEC-C migration to that of shape change releasate levels (49.5±4.7 vs. 55.7±4.0 cells per field of view, *P>0.05*) (Fig. 3).

### Anti-platelet Factors Prevent Angiostatin Release

**Figure 4:**
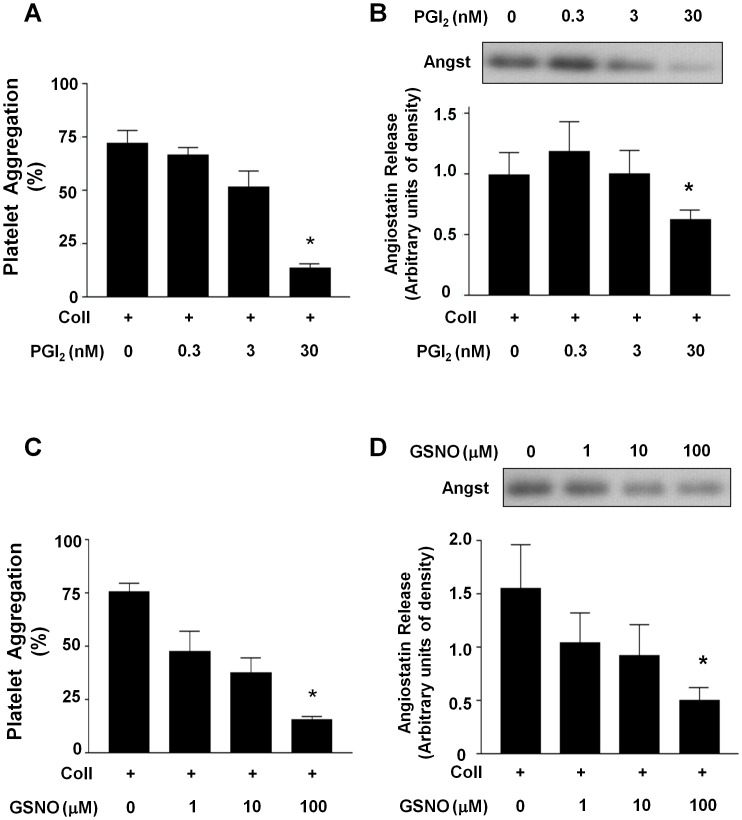
Effect of physiological platelet inhibitors on angiostatin release. (**A**) Summary data demonstrating the concentration inhibitory effects of PGI_2_ on platelet aggregation. (**B**) Representative immunoblot and summary densitometry data demonstrating the inhibitory effects of PGI_2_ on platelet angiostatin release. (**C**) Summary data demonstrating the concentration inhibitory effects of the nitric oxide donor GSNO on platelet aggregation. (**D**) Representative immunoblot and summary densitometry data demonstrating the inhibitory effects of GSNO on platelet angiostatin release. Collagen (3 µg/ml) was used to induce aggregation. N = 5. *, P<0.05 vs. control. Angst – Angiostatin.

**Figure 5:**
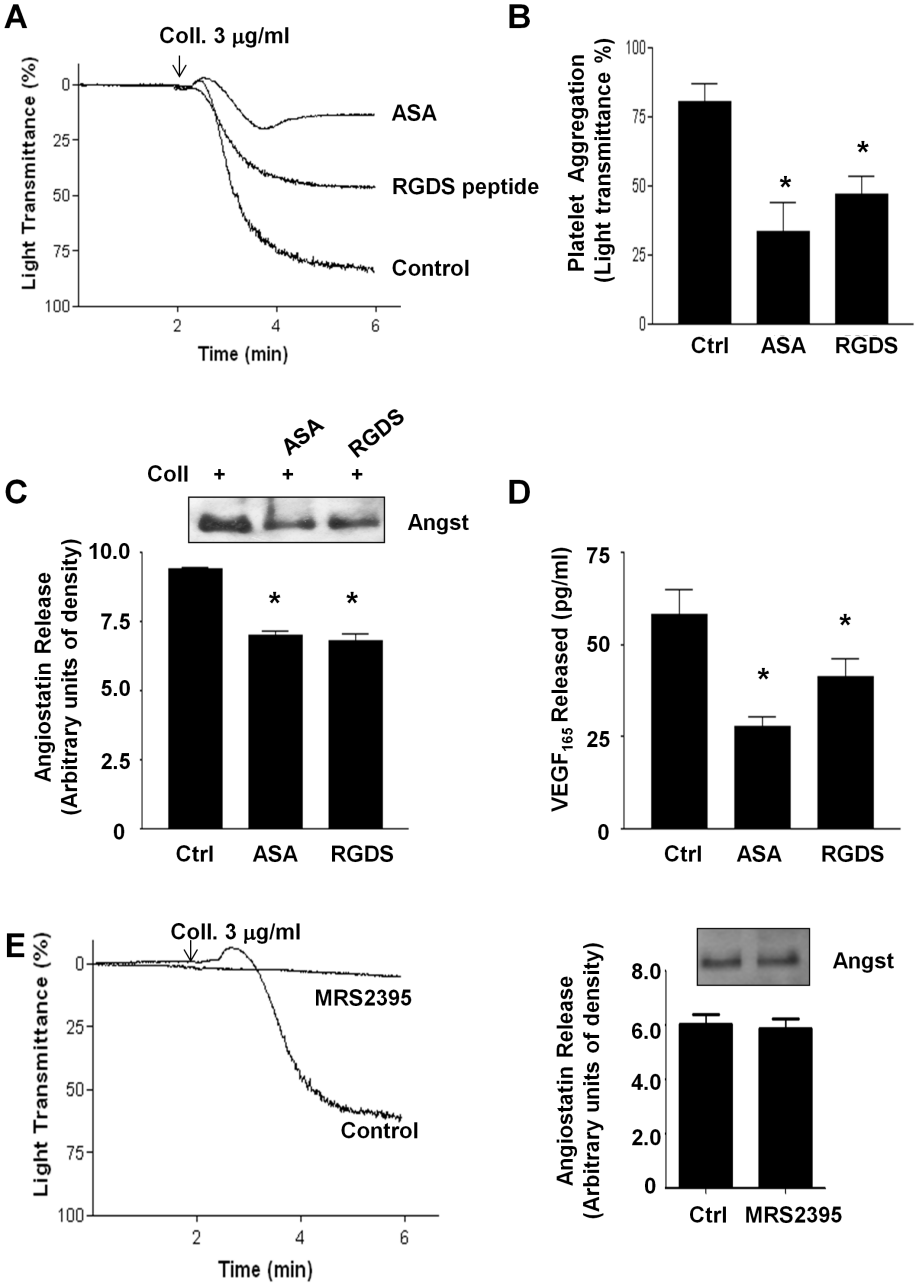
Effect of anti-platelet agents on angiostatin release. (**A**) Represenative traces and (**B**) summary data demonstrating the inhibitory effects of ASA (100 µM) and RGDS (10 µM) on platelet aggregation. (**C**) Represenative immunoblot and summary densitometry data demonstrating the inhibitory effects of ASA (100 µM) and RGDS (10 µM) on platelet angiostatin release. (**D**) Summary data demonstrating the inhibitory effects of ASA (100 µM) and RGDS (10 µM) on platelet VEGF release. (**E**) Represenative traces demonstrating the inhibitory effects of MRS2395 (50 µM) on platelet aggregation. (**F**) Represenative immunoblot and summary densitometry data demonstrating that MRS2395 does not inhibit angiostatin release. N = 4. *, P<0.05 vs. control. Angst – Angiostatin.

Because platelet aggregation and thrombus formation are inhibited by physiological factors such prostacyclin (PGI_2_), nitric oxide (NO) and by pharmacological anti-platelet agents [23], we investigated the effects of platelet inhibition on angiostatin release. Both prostacyclin and the NO-donor s-nitroso-glutathione (GSNO) caused a concentration-dependent decrease in angiostatin release from human platelets (Fig. 4A–D). The common anti-platelet drug acetylsalicylic acid (100 µM) and a glycoprotein IIb/IIIa (GPIIb/IIIa) blocking peptide RGDS (10 µM) both significantly inhibited platelet aggregation and angiostatin release (Fig. 5A–C). These two pharmacological agents also suppressed platelet VEGF release (Fig. 5D). Interestingly, blockade of the platelet ADP P2Y12-receptor failed to prevent angiostatin release from platelets in light of potent platelet-aggregation inhibition (Fig. 5E).

### Anti-platelet Agents Prevent Angiogenesis Regulator Release from Platelet α-granules but not De Novo Angiostatin Generation

**Figure 6:**
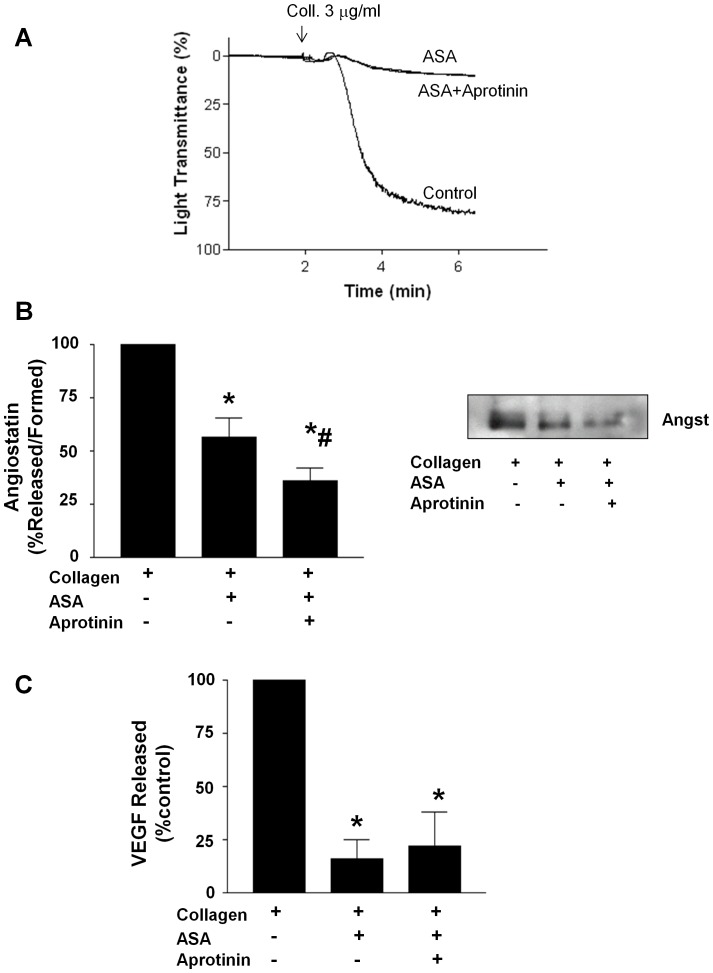
ASA suppresses platelet angiostatin release but not generation. (**A**) Represenative traces demonstrating the effects of ASA (100 µM) and aprotinin (30 µM) on platelet aggregation. (**B**) Summary densitometry data and represenative immunoblot demonstrating the effects of ASA and aprotinin on platelet angiostatin generation/release. **(C)** Summary data demonstrating the effects of ASA and aprotinin on platelet VEGF release. N = 4. *, P<0.05 vs. control. Angst – Angiostatin.

Since angiostatin in addition to being released from platelets is also constitutively generated on the platelet surface membrane in a uPA-dependent manner [9], we investigated whether the prototypical anti-platelet agent acetylsalicylic acid (ASA) could prevent *de novo* angiostatin generation also. Although ASA inhibited angiostatin release from platelets, aprotinin a serine protease inhibitor previously shown to inhibit *de novo* platelet angiostatin generation [9] further decreased angiostatin levels in collagen-aggregated releasates. Thus, ASA did not prevent angiostatin generation during aggregation (Fig. 6A–B). Importantly, aprotinin had no effect on collagen-induced platelet aggregation or VEGF release (Fig 6A & C).

**Figure 7:**
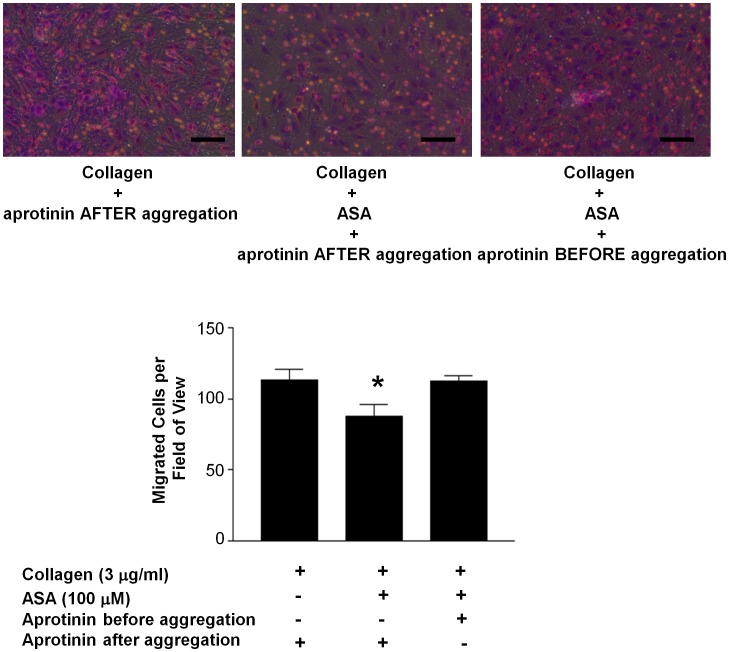
Suppression of platelet angiostatin generation enhances endothelial cell migration in the presence of ASA. Representative photomicrographs and summary data demonstrating the inhibitory effects of ASA (100 µM) on platelet-stimulated HMVEC-C migration under hypoxic conditions. Inhibition of platelet angiostatin generation by aprotinin (30 µM) reverses the migration inhibitory effects of ASA-inhibited platelet releasates. Platelets were aggregated by collagen (3 µg/ml). ASA was added to platelet rich plasma during platelet preparation and then subsequently washed away. An equal concentration of aprotinin was added to control and ASA-treated platelet releasates following aggregation. This was done to account for any potential affects of aprotinin in the releasates on the migration assays. Scale bars = 100 µm. N = 4. *, P<0.05 vs. control.

To determine the potential functional significance of blocking platelet angiostatin generation by aprotinin in the presence of ASA on angiogenesis, we once again performed endothelial cell migration assays under hypoxic conditions. Compared to control collagen-aggregated platelet releasates, those isolated from ASA-inhibited platelets promoted HMVEC-C migration to a lesser extent (113.1±7.3 vs. 87.8±8.2 cells per field of view, *P<0.05*) (Fig. 7). However, ASA-inhibited platelets that were simultaneously incubated with aprotinin to inhibit angiostatin generation during aggregation restored endothelial cell migration to control levels (113.1±7.3 vs. 112.5±3.1 cells per field of view, *P>0.05*) (Fig. 7)

## Discussion

The major novel finding of our study is that there is a temporal difference in the release of pro- (VEGF) versus anti-angiogenesis (angiostatin) regulating factors from platelets upon aggregation. This temporal difference in angiogenesis regulator secretion has the potential to significantly impact angiogenesis in hypoxic cellular microenvironments following thrombus formation. Indeed, our results show that the platelet release reaction during the early stages of activation (platelet shape change) is more potent at promoting endothelial cell migration under conditions of low oxygen than following complete (maximal) aggregation. This differential temporal angiogenesis regulator secretion is likely possible due to segregation of VEGF and angiostatin into distinct α-granule subsets or α-granule sub-compartments. Previous studies have shown that pro- and anti-angiogenesis regulators such as VEGF and endostatin are segregated within α-granules and differentially released via proteinase-activated receptor (PAR)-signalling [20], [22]. Recent evidence suggests that α-granule proteins are packaged in distinct zones, vesicles, or microparticles [21], [24], which would be consistent with our finding of little/no VEGF and angiostatin co-localization. Importantly, we show that angiostatin is responsible for the endothelial cell migration tempering effect of releasates derived from maximally aggregated platelets, as its neutralization fully restored endothelial cell migration under hypoxic conditions. The full consequence of this finding still needs investigating, but it has been speculated that physiologically platelet-derived angiostatin may contribute to down-regulation of angiogenesis at the end stages of wound healing [25]. Although released minutes apart from platelets, VEGF and angiostatin may initiate a series of temporally different signals within endothelial cells that help coordinate the wound healing response over the course of days. Similarly, platelet-derived angiostatin may be a potent factor limiting therapeutic neo-vascularisation following pathological thrombus formation.

As the endothelium generates NO and prostacyclin, which prevent platelet adhesion and aggregation [23], [26], we investigated the effect of these two molecules on platelet angiostatin release. Both NO and prostacyclin reduced platelet aggregation and consequently angiostatin release from α-granules. The findings are in line with previous studies that have demonstrated that these two endogenously generated molecules concomitantly inhibit platelet aggregation and α-granule release [27], [28].

In addition, to molecules that can be generated endogenously, we investigated the effects of common anti-platelet agents. Anti-platelet drugs prevent aggregation/thrombus formation and consequently can influence platelet-stimulated angiogenesis. Hence, we investigated their effect on angiostatin release and endothelial migration. We chose inhibitors that blocked the most commonly clinically targeted platelet enzymes and receptors, including ASA, which blocks platelet cyclooxygenase. We used MRS2395 to antagonize platelet P2Y12 receptors in place of clinically used clopidogrel or prasugrel, as these two pro-drugs require hepatic biotransformation to active metabolites. Finally, we used a GPIIb/IIIa blocking peptide, RGDS, in place of clinically used GPIIb/IIIa antagonists such as Abciximab™. ASA and the GPIIb/IIIa blocking peptide concomitantly inhibited collagen-induced platelet aggregation, angiostatin, and VEGF release. ASA has been previously shown to inhibit ADP- and thrombin (albeit weakly)- induced VEGF release from platelets [29], [30], while Abciximab™ has been shown to suppress VEGF release from tumor cell aggregated platelets [31]. Hence, blockade of platelet cyclooxygenase activity or GPIIb/IIIa fibrinogen binding suppresses release of both pro- and anti-angiogenesis regulating factors from platelet α-granules or α-granule sub-compartments. The finding that P2Y12 blockade by MRS2395 failed to suppress angiostatin release is consistent with other studies also. Although P2Y12 antagonism prevents platelet VEGF release [29], its antagonism by ticlopidine increases the release of endostatin, another angiogenesis inhibitor, in spite of platelet aggregation suppression [30]. This differential effect of COX versus P2Y12 antagonism on pro- vs. anti-angiogenesis regulator release from platelet α-granules may have important consequences on neo-vascularisation and therapeutic angiogenesis. Whether the choice of anti-platelet agent can influence the growth of new blood vessels to ischemic tissues will need to be investigated.

With enhancement of neo-vascularisation in mind, we further investigated whether ASA would also suppress angiostatin generation by platelets in addition to its release. We found that although ASA inhibited angiostatin release from α-granules, it did not suppress *de novo* angiostatin generation, as the serine protease inhibitor aprotinin further reduced angiostatin levels in platelet releasates. Aprotinin had no inhibitory effect on platelet aggregation and VEGF release in response to collagen. However, by blocking angiostatin generation, aprotinin restored endothelial cell migration in response to ASA-inhibited platelet releasates to levels induced by control collagen-aggregated releasates. Thus, in the presence of platelet inhibition, inhibiting angiostatin generation or its neutralization may be beneficial to therapeutic angiogenesis in hypoxic microenvironments following thrombus formation. Aprotinin, an anti-fibrinolytic previously widely used to reduce bleeding following cardiac surgery, may be a candidate agent to help promote neo-vascularisation by inhibiting angiostatin generation. Although, the use of aprotinin as an anti-fibrinolytic agent was suspended following early termination of the BART trail over concerns of increased mortality [32], recent meta-analysis have shown aprotinin to be safe and effective [33].

In summary, we have found that platelets release VEGF earlier during aggregation than angiostatin and that this temporal difference in angiogenesis regulator release results in a differential ability to stimulate endothelial cell migration. This temporal difference in pro- vs. anti-angiogenesis regulator release from platelets may help to explain how platelets, in spite of containing angiogenesis regulators with opposing actions, can effectively coordinate angiogenesis during wound healing. Furthermore, we found that in addition to PGI_2_ and NO, anti-platelet agents that block COX or GPIIb/IIIa suppress platelet angiostatin release but not its generation. We propose that in the presence of anti-platelet agents direct inhibition of angiostatin or its generation may enhance platelet-stimulated angiogenesis. Moreover, the neutralization of an angiogenesis inhibitor may also enhance therapeutic angiogenesis strategies that to date have strictly focused on pro-angiogenic protein, genes, or cells. Thus, we propose that by combining angiostatin neutralization with anti-platelet therapy the dual beneficial effect of improved therapeutic angiogenesis in ischemic tissue and decreased risk of thrombotic events may be achieved.

## Supporting Information

Figure S1 Representative photomicrographs and summary data of endothelial cell migration under normoxic conditions toward a platelet releasate gradient. Releasates from collagen-aggregated platelets (maximum aggregation time point) provide a stronger stimulus for endothelial cell migration than resting (non-activated) platelet releasates. Summary data from two independent experiments performed in duplicate. Scale bars indicate 100 µm.
(PDF)
