# Supplementary material for: Temporal and Pharmacological Characterization of Angiostatin Release and Generation by Human Platelets: Implications for Endothelial Cell Migration
Source: PLoS One. 2013 Mar 15;8(3):e59281. doi: 10.1371/journal.pone.0059281 (PMC3598756; doi:10.1371/journal.pone.0059281)
Supplement: Figure S1 — Representative photomicrographs and summary data of endothelial cell migration under normoxic conditions toward a platelet releasate gradient. Releasates from collagen-aggregated platelets (maximum aggregation time point) provide a stronger stimulus for endothelial cell migration than resting (non-activated) platelet releasates. Summary data from two independent experiments performed in duplicate. Scale bars indicate 100 µm. (PDF) [file pone.0059281.s001.pdf]

Radziwon-Balicka et al., **Temporal and Pharmacological Characterization of Angiostatin Release and Generation by Human Platelets: Implications for Endothelial Cell Migration,** PLOS One

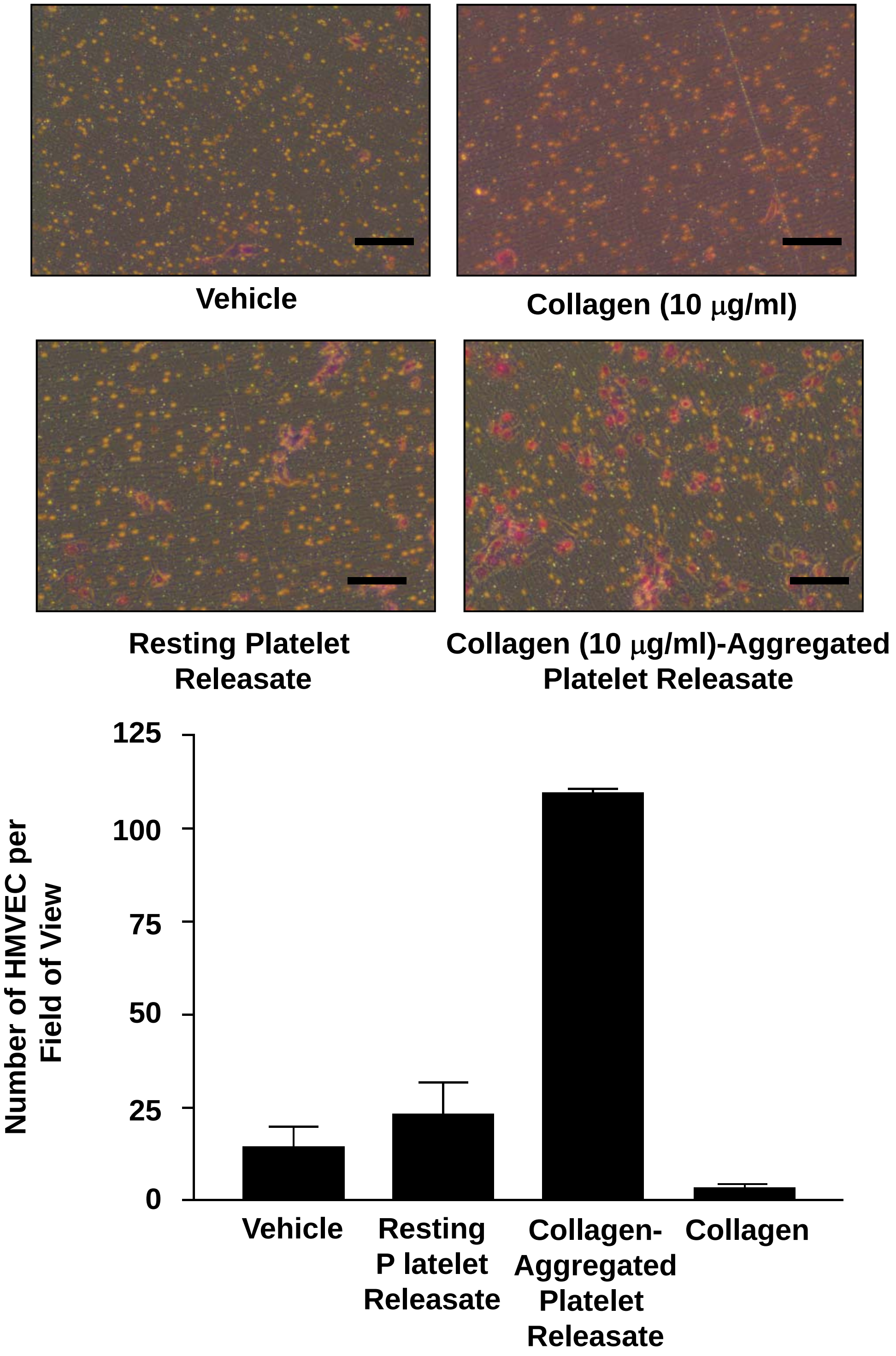

Supplemental Data Figure 1. Representative photomicrographs and summary data of endothelial cell migration under normoxic conditions toward a platelet releasate gradient. Releasates from collagen-aggregated platelets (maximum aggregation time point) provide a stronger stimulus for endothelial cell migration than resting (non-activated) platelet releasates. Summary data from two independent experiments performed in duplicate. Scale bars indicate 100 μm.
